# Within-person adaptivity in frugal judgments from memory

**DOI:** 10.1007/s00426-017-0962-7

**Published:** 2017-12-22

**Authors:** Elisa Filevich, Sebastian S. Horn, Simone Kühn

**Affiliations:** 1 Center for Lifespan Psychology, Max Planck Institute for Human Development, Lentzeallee 94, 14195 Berlin, Germany; 2 Department of Psychology, Humboldt-Universität zu Berlin, Berlin, Germany; 3 Bernstein Center for Computational Neuroscience, Berlin, Germany; 4 Berlin School for Mind and Brain, Berlin, Germany; 5 Center for Adaptive Rationality (ARC), Max Planck Institute for Human Development, Berlin, Germany; 6 Department of Psychology, University of Zurich, Zurich, Switzerland; 7 Klinik und Poliklinik für Psychiatrie und Psychotherapie, Universitätsklinikum Hamburg-Eppendorf, Hamburg, Germany

## Abstract

Humans can exploit recognition memory as a simple cue for judgment. The utility of recognition depends on the interplay with the environment, particularly on its predictive power (validity) in a domain. It is, therefore, an important question whether people are sensitive to differences in recognition validity between domains. Strategic, intra-individual changes in the reliance on recognition have not been investigated so far. The present study fills this gap by scrutinizing within-person changes in using a frugal strategy, the recognition heuristic (RH), across two task domains that differed in recognition validity. The results showed adaptive changes in the reliance on recognition between domains. However, these changes were neither associated with the individual recognition validities nor with corresponding changes in these validities. These findings support a domain-adaptivity explanation, suggesting that people have broader intuitions about the usefulness of recognition across different domains that are nonetheless sufficiently robust for adaptive decision making. The analysis of metacognitive confidence reports mirrored and extended these results. Like RH use, confidence ratings covaried with task domain, but not with individual recognition validities. The changes in confidence suggest that people may have metacognitive access to information about global differences between task domains, but not to individual cue validities.

**Electronic supplementary material:**

The online version of this article (10.1007/s00426-017-0962-7) contains supplementary material, which is available to authorized users.

## Introduction

Organisms have to make predictions and inferences in an inherently uncertain world. An influential perspective on judgment and decision making suggests that humans achieve this by relying on available cues (pieces of information) that are only probabilistically related to some criterion in the environment (Brunswik, 1952). For instance, physicians consider specific symptoms to make their diagnoses, judges decide whether to release a defendant on bail based on the past records (Dhami, Hertwig, & Hoffrage, 2004), and consumers might infer the quality of products based on the price or by recognizing their brand names (Rao & Monroe, 1989). Importantly, such cues are not universally useful across different domains of decision making. In this article, we focus on the individual ability to use recognition memory adaptively as a cue across different situations.

Recognition memory (i.e., the ability to discriminate between familiar and novel items) provides a particularly simple cue for inference that is retrieved rapidly and with little effort (e.g., Rosburg, Mecklinger, & Frings, 2011). Goldstein and Gigerenzer (2002) found that people frequently utilize recognition for their judgments and specified a model—the *recognition heuristic* (RH)—that explains people’s strategic inferences when two items must be ordered along a quantitative criterion (e.g., inferring which of two cities is more populous). According to the RH, if one item is recognized (e.g., Prague) and the other is not (e.g., Erdenet), then the recognized item is inferred to have the higher value on the criterion dimension (on which the objects’ true values are unknown). The RH has been extensively investigated (e.g., Goldstein & Gigerenzer, 2002; Hilbig, Michalkiewicz, Castela, Pohl, & Erdfelder, 2015; Kämmer, Gaissmaier, Reimer, & Schermuly, 2014; McCloy, Beaman, Frosch, & Goddard, 2010; Newell & Shanks, 2004; Pachur, Bröder, & Marewski, 2008) and is a prime example of a frugal heuristic that exploits one good reason (recognition) that leads to surprisingly accurate judgments in many real-world domains because familiar and novel items often differ systematically on relevant dimensions such as quantity or success (e.g., sports teams, brands, stocks, and colleges that are recognized tend to be more successful). Even though people may not always give recognition primacy over other information in strict non-compensatory fashion, as originally specified for the RH (Glöckner, Hilbig, Jekel, 2014; Hilbig et al. 2015; Newell & Shanks, 2004), research on how recognition is systematically exploited as a cue remains important (for overviews, see Marewski, Pohl, & Vitouch, 2010; Pachur, Todd, Gigerenzer, Schooler, & Goldstein, 2011).

As any other rule of thumb, the RH is not a bad or good strategy per se: its success depends on the interplay with the environment, that is, the fit to the problem at hand (Gigerenzer & Goldstein, 2011). For a given domain or task, the judgment accuracy attainable with the RH can be quantified as *recognition validity* (*α*), which measures the strength of covariation between recognition memory and the criterion dimension.^1^ Whereas recognition is often a valid cue for inferences of properties of real-world items (Goldstein & Gigerenzer, 2002; Pohl 2006), ecological analyses indicate that whenever the frequency of mentions by mediators in the environment (e.g., by other people, electronic media, newspapers, etc.) does not uniquely map onto a relevant criterion dimension, then the RH tends to fare poorly (see Pachur et al. 2011). For instance, the recognition validity α is relatively low for inferring the frequency of diseases in a country (Pachur & Hertwig, 2006) or the distance of cities to an arbitrary geographical reference point (Pohl 2006). Following these considerations, a central question surrounding research on the RH is to what extent it is used adaptively (Gigerenzer & Goldstein, 2011; Pachur et al. 2011; Pohl 2006; Pohl, Michalkiewicz, Erdfelder, & Hilbig, 2017). That is, how sensitive are people to differences in recognition validity and do they adaptively adjust their reliance on the RH accordingly?

## Adaptive reliance on recognition

In a review on the RH, Gigerenzer and Goldstein (2011) explored the relation between RH use and the recognition validity across various domains. Pooling the data from 43 studies and domains yielded a positive linear relation of *r* = .57 between the mean frequency of judgments in accordance with the RH and participants’ average recognition validity. Beyond these cross-study correlations, only few investigations addressed this issue experimentally and directly compared participants’ adaptive RH use between task domains that differed systematically in the validity of recognition: Pohl (2006) found that name recognition of Swiss cities predicted their population (*α* = 0.86), but not their distance to a geographical reference point, the city Interlaken (*α* = 0.51). Correspondingly, participants relied more on recognition^2^ in inferences of the city populations (0.66) than of the distances (0.08). Similarly, Hilbig, Erdfelder and Pohl (2010; Exp. 7) found that people relied more on recognition (0.65 vs. 0.20) when making inferences of the population of Italian cities (*α* = 0.87) than of their height above sea level (*α* = 0.53). Data from Pachur, Mata, and Schooler (2009) also indicated that people’s reliance on recognition was substantially higher (0.80 vs. 0.30) in a domain with high-recognition validity (inferences of US city populations; *α* = 0.90) than in a domain with low-recognition validity (inferences of the frequency of diseases; *α* = 0.62). These findings suggest that the *group averages* of RH use closely match the mean recognition validity in a given domain and that recognition validity is a central factor explaining how frequently the RH is applied. However, it is still an open question whether and how single individuals adaptively adjust their reliance on recognition. In the literature, two different interpretations have been discussed how adaptive RH use may come about.

One possibility is that individuals are sensitive to the validity of their own recognition knowledge and use the RH in a proportion of trials that matches this validity (henceforth termed *matching hypothesis*; see also Pachur & Hertwig, 2006). However, little support emerged for this notion so far, as correlations between RH use and α within a specific domain have been found to be negligible. For instance, both Pachur and Hertwig (2006) and Pohl (2006) observed that individual proportions of judgments in accordance with the RH were uncorrelated with the individual α within a given domain. Moreover, there is evidence that people’s estimates of the validity of their own recognition knowledge are not very accurate (Pachur et al. 2008). In this vein, Pohl et al. (2017) investigated whether use of the RH is influenced by the validity of the specific set of selected items or by the underlying domain from which these items were sampled (global domain validity). Importantly, their findings indicated that it is the validity of the general environment (rather than that of specific items) that impacts strategy use, suggesting that participants behave as if all items were approximately representative of an underlying domain. Hence, as an alternative possibility, people may notice cue-validity differences between task domains on a more global level and adjust their RH use accordingly (henceforth termed *environment adaptivity hypothesis*); in this case, adaptive changes in strategy use may result from having fuzzier intuitions about the usefulness of a cue in a given domain rather than considering (or even computing) individual cue validities. Even though these intuitions may not be perfect, they could nonetheless foster adaptive decision-making and robustly capture rank differences in cue validities across tasks (Wright & Murphy, 1984; see Katsikopoulos, Schooler, & Hertwig, 2010, for further discussion), as long as items are representatively sampled (Pohl et al., 2017).

Taken together, the notion of adaptive selection of the RH (or any other strategy) implies dynamic within-person changes in behavior. Crucially however, the aforementioned hypotheses about adaptive RH use have never been tested *within individuals*, to the best of our knowledge. This is important for several reasons. The first reason is conceptual: when a theory proposes individual, adaptive change, then these assumptions should be put to test with data that correspond as closely as possible. We, therefore, note a mismatch between the theoretical proposition of individual adaptivity and data from previous analyses, which examined variability *between* individuals. Second, and apart from usual methodological considerations (such as reductions in unsystematic variance through repeated testing of the same participants), experiments in various fields of psychology have shown that effects of independent variables are often different in within- and between-subjects designs (Erlebacher 1977). The generalizability across designs hinges on the assumption of variation equivalence (i.e., the assumption that processes generating variability within and between individuals are identical; Lindenberger & von Oertzen, 2006). Moreover, situations are conceivable in which mean-level changes are produced by only a minority of subjects (as reflected, e.g., in the conceptual distinction between differential and mean-level and stability in personality research; e.g., Josef et al. 2016). Finally, some aspects of strategy use can only be tested within participants. For example, can we observe some stability in the reliance on recognition (e.g., rank-order stability), even when the validity of a cue changes dramatically?

In this study, we addressed these issues and set out to investigate adaptive use of the RH within the same decision-makers across two task domains that strongly differed in their recognition validity, using a modeling approach that provides estimates of individual parameters as well as their correlations. In addition, we aimed to gain further insight into people’s adaptive RH use by considering their metacognitive reports.

## Metacognitive monitoring and use of recognition

Metacognitive monitoring refers to the human ability to introspect and report one’s own mental states (e.g., to evaluate how much we know; Koriat, 2007). Confidence reports can be viewed as a central aspect of metacognitive monitoring (Fleming, Dolan, & Frith, 2012). In this study, we considered judgment confidence as an additional measure for understanding adaptive strategy use and asked participants, after each trial, about their confidence in the correctness of their preceding judgment. Historically, research on the RH originated from investigations of subjective confidence and the question of when people exhibit overconfidence (Fischhoff, 1982) in their judgment accuracy (Hoffrage, Hafenbrädl, & Marewski, 2017).^3^ Notwithstanding these important considerations, we focused here on the *relative change* in confidence as a function of task environment and the relation with strategy use. We expected that people’s confidence should be influenced by the ecological validity of the most relevant cue in probabilistic inference problems (Gigerenzer, Hoffrage, & Kleinbölting, 1991) and should thus change as function of task domain.

For the present analyses, we borrowed from research on signal detection the notion of *metacognitive sensitivity* that refers to the degree to which peoples’ confidence tracks their performance (Fleming & Lau, 2014). Metacognitive sensitivity is dissociable from accuracy in a task (e.g., the proportion of correct judgments) and from setting a subjective criterion (i.e., the strength of internal evidence required to report a certain level of confidence; for further details, see Fleming & Lau, 2014). First, the distinction between metacognitive sensitivity and task accuracy acknowledges the possibility that participants may commit many errors in a task, but are nevertheless highly sensitive to their performance and assign high confidence only to their few correct responses (and rarely to their incorrect responses). Second, this implies that confidence reports are a function of two separable components, the strength of an internal signal and a subjective criterion. One approach to measure metacognitive sensitivity is to calculate the slope parameter for each participant in a regression model that provides an estimate for the relationship between confidence and judgment accuracy. Large slope estimates would indicate a strong relationship between the two variables and high metacognitive sensitivity: Small differences in accuracy are readily detected in metacognitive monitoring and result in significant differences in confidence. Conversely, slope estimates close to zero would indicate poor metacognitive sensitivity and imply that even larger differences in accuracy do not result in significant changes in confidence (Norman, Price & Jones, 2011; Sandberg et al. 2010). Modeling confidence in this way helps us to interpret people’s metacognitive reports between tasks: If participants show similar metacognitive sensitivity across two experimental conditions (i.e., a consistent relationship between confidence and accuracy), then any potential differences in confidence reports cannot be attributed to poorer metacognitive insight in one of the conditions. Consequently, the absolute values of confidence ratings can be directly compared in such a situation. Differences in confidence ratings between two conditions would then support the conclusion that these conditions have different properties (e.g., different cue validities) that are accessible on a metacognitive level. In this study, we used this approach to interpret people’s confidence reports in the comparative judgment task.

Both the matching and the environment adaptivity hypotheses are silent about the relation between introspective experience and adaptivity and neither hypothesis makes explicit predictions about confidence ratings. We argue, however, that confidence reports could be used to further refine and test these predictions. When participants follow recognition as a cue, its validity could have an impact on subsequent confidence ratings. That is, a cue of higher validity would more frequently lead to a correct judgment than a less valid cue; individuals with higher recognition-cue validities (and metacognitive access to them) could, place higher confidence in judgments following recognition (and vice versa for individuals with lower recognition validities). Therefore, if people are sensitive to the individual validity of recognition, as the matching hypothesis suggests, their confidence in decisions that follow the RH should correlate with their individual cue validities. In the absence of such a correlation, however, one could conclude that people have little metacognitive access or intuition about their recognition validities, which would provide further evidence against the matching hypothesis. In contrast, the environment adaptivity hypothesis implies that if people had metacognitive access to the validity of recognition across different domains, then their mean confidence ratings should also differ between these task domains. In the following, we tested these predictions about RH use and associated confidence reports using a within-subjects design.

## Method

### Participants and recruitment

Ninety-nine participants (48 female; age *M* = 36.6 years; *SD* = 11.2; range 20–69), recruited through Amazon’s Mechanical Turk service (Crump et al. 2013), completed this study via the Internet. Data were collected on a private institutional server managed by the JATOS tool (Lange, Kühn, & Filevich, 2015).^4^ Informed consent was obtained from each participant included in the study. All participants reported to be citizens and residents of the USA at the time of participation, and were informed that they could quit the study at any point. The study was announced to last for 30 min and remuneration for participation was set at 3 USD. Participants who did not complete the entire study were not remunerated. Average completion time for the study was 28 min. All procedures were approved by the local ethics committee.

### Procedures and design

**Fig. 1:**
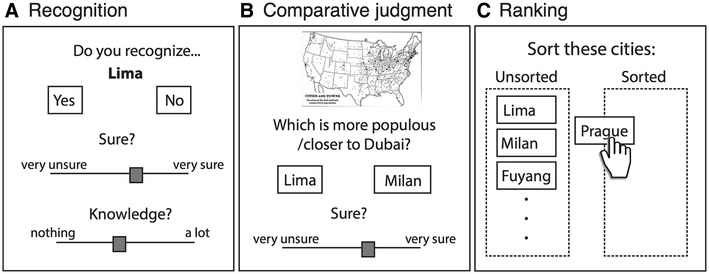
Tasks used in the study. Each participant completed three tasks. **a** Recognition task, in which participants reported whether they had heard of a given city before, how confident they were of recognizing it, and how much further knowledge they had about the city. **b** Comparative judgment task, in which participants chose, for each of 70 pairs, which of two cities was more populous (population domain) or closer to a geographical reference point (distance domain). Participants additionally indicated their confidence in their own responses. **c** A ranking task, in which participants sorted cities along their estimated values on the criterion dimension

Following standard procedures in research on the RH, all participants completed a *recognition task* (Fig. 1a) and were asked to indicate whether they had heard or seen the name of a certain city before. Participants saw the name of each of 16 cities, displayed in random sequence on the screen and indicated with a key press whether they recognized it. After each recognition judgment, participants additionally provided ratings on visual analog scales about their confidence in their own recognition judgment and how much further knowledge they had about the given city. The recognition test also included one city name (Dubai) that was used as geographical reference point for the distance domain of the inference task; we selected this city because it was recognized by most participants in pilot testing and the distribution of distances between this and the other cities showed sufficient variability; 100% of the participants in our main study reported to recognize the city of Dubai. Table 1 shows a list of all city names presented in this study, their corresponding criterion values (i.e., city populations and distances), and participants’ recognition rates. In a *comparative judgment task* (Fig. 1b) participants judged on each trial which one in a pair of city names had the higher criterion value. After each judgment, participants additionally indicated their confidence in their response to the comparative judgment on a visual analog scale. Each participant completed two different domains of the judgment task: In the *population domain*, participants indicated which of two cities had the larger population (70 trials); in the *distance domain*, participants indicated which of two cities was located closer to the city of Dubai (70 trials). We chose these two domains because they are expected to differ strongly in recognition validity (for a previous application, see Pohl 2006) and make it possible to test people’s adaptivity while simultaneously holding stimulus materials (including recognition rates and proportion of cases where the RH can be applied) constant.

**Table 1:** Stimulus materials used in the inference task and recognition rates

City name	% of participants recognizing the item	Mean reported further knowledge^a^	Confidence in recognition judgment^a^	Population criterion^b^	Distance criterion^c^
Istanbul	97.98	40.79	96.75	13,000,000	2994
Seoul	96.97	43.5	96.43	10,000,000	6783
Munich	95.96	41.75	96.81	1,400,000	4567
Prague	94.95	36.62	95.78	1,240,000	4446
Milan	93.94	39.77	93.72	5,200,000	4663
Cape Town	92.93	34.57	94.45	3,000,000	7642
Lima	88.89	29.63	92.15	7,500,000	14,813
Oslo	81.82	30.33	93.05	634,000	5138
Vilnius	11.11	5.86	86.03	535,000	4091
Malindi	10.1	5.06	81.68	207,000	3666
Vientiane	9.09	5.93	87.09	783,000	4934
Changsha	8.08	4.34	87.24	7,000,000	5685
Ouagadougou	5.05	4.36	86.21	1,600,000	6106
Fuyang	4.04	2.91	81.08	1,700,000	5073
Erdenet	3.03	4.73	89.93	86,000	4955

^a^Scaled from 0 to 100

^b^Number of inhabitants (retrieved from http://www.wikipedia.org)

^c^Flying distance to Dubai in km (retrieved from http://www.distancecalculator.globefeed.com)

In a final *ranking task* (Fig. 1c), participants saw the list of preceding cities in random arrangement (displayed on interactive drag-and-drop HTML element) and were asked to sort them along to their estimated population and distance values. Each participant completed this task twice: once for the population-size criterion and once for the distance criterion.

The order of the recognition task and comparative judgment task and the order of the population and distance domains were counterbalanced between subjects. Each participant completed the comparative judgment twice, once for the population and once for the distance domain.^5^

Participants were instructed to carefully read and answer each question and to provide their best possible judgments. Moreover, participants were informed that we recorded their response times (RTs) and whether and when they left the browser window or tab during the study (e.g., by clicking elsewhere).

### Stimulus materials

We used the most common stimulus material for investigating the RH, the names of real-word cities. Sixteen city names were selected from a larger pool. As mentioned above, one name (Dubai) served as geographical reference point for the distance domain; the remaining 15 names were used for the comparative judgment task. For each participant, 70 pairs of city names (out of a total of 105 possible pairings) were randomly selected for the comparative judgment task; for a given participant, this same set of 70 pairs was then used in all experimental domains. Halfway through data collection, we mirrored the screen position of the city names in the pairs (i.e., for half of the participants, city names displayed on the right side of the screen were now displayed on the left side, and vice versa).

### Measurement-modeling approach

To measure participants’ heuristic reliance on recognition, we used a multinomial processing tree (MPT) modeling approach that makes it possible to evaluate goodness-of-fit and to conduct model comparisons (see Batchelder & Riefer, 1999; Erdfelder et al. 2009, for overviews). The choice of a recognized item in the comparative judgment task can result from reliance on the recognition cue, but also from guessing, or the use of other cues (or knowledge) that are associated with the criterion. In consequence, the proportion of judgments in which people choose the recognized item is a confounded measure that can overestimate RH use (Hilbig et al. 2010). In this study, we used the MPT *r*-model because it effectively disentangles reliance on recognition (as assumed by the RH) from the use of further knowledge (or any other strategy). Specifically, the *r*-model provides probability estimates for the reliance on recognition (parameter *r*), for the cue validity of further knowledge, and of recognition. Further details are in “Appendix A” (Hilbig et al. 2010; Horn et al. 2015, 2016; we also explored a memory-state model extension that did not alter the main conclusions in the present study; see Castela, Kellen, Erdfelder, Hilbig, 2014).

For our analyses, we implemented a hierarchical latent-trait version of the *r*-model (Klauer 2010; Matzke, Dolan, Batchelder, & Wagenmakers, 2015) that accounts for diversity in strategy use and that circumvents aggregation over individuals (see Siegler 1987, for a critical discussion of pooling over subjects for investigations of strategy use). We adjusted the model to simultaneously account for the two within-subject conditions (domains) of the study. One advantage of this approach is that overarching group-level distributions constrain the individual estimates in a theoretically principled way and may thus yield more reliable individual parameters. Importantly, the latent-trait approach makes it possible to jointly estimate model parameters as well as their correlations. By adjusting for the uncertainty of the individual estimates, this approach promises to avoid the potential biases involved in computing standard Pearson correlations of individual estimates and to provide an assessment of these relations that is decontaminated from error influences (Klauer 2010).

Estimation of the model parameters relied on a Bayesian approach (see Lee & Wagenmakers, 2013, for an overview), which has been employed in numerous areas of cognitive modeling (e.g., Kellen, Pachur, Hertwig, 2016; Nunez, Srinivasan, & Vandekerckhove, 2015; Steingroever, Pachur, Šmíra, & Lee, 2017; Thiele, Haaf, & Rouder, 2017). To determine the most credible value ranges of the model parameters in the posterior distributions given the data, we used the Markov chain Monte Carlo (MCMC) methodology for posterior sampling,^6^ Further details of the hierarchical implementation and the prior distributional assumptions are in “Appendix B” (cf. Heck, Arnold, & Arnold, 2017; Matzke et al. 2015). We report posterior means with 95% Bayesian-credible intervals. Specifically, *μ*_*r*POP_ and *μ*_*r*DIS_ refer to the mean group-level estimates of reliance on recognition in the Population and Distance domain, respectively; moreover, we report the standard deviations (*σ*_*r*POP_, *σ*_*r*DIS_) and the correlations (*ρ*) among the model parameters. The standard deviations represent the variability between participants and are close to zero when participants are homogeneous and are large in the case of substantial diversity.

### Data quality checks

As data were collected online, we first examined indicators of the quality of these data. We found no evidence that the data collected online were suspect of careless responding, attentional lapses, or sequence effects. However, a few participants left the browser tab during the experiment, as described below.

#### Focus on the task

We monitored, on each trial during the experiment, whether participants clicked anywhere outside the browser tab (e.g., potentially left the task for a short period of time). We then examined the frequencies of these “lost-focus events” for each participant and domain. Notably, participants rarely left the experiment tab. In the recognition task, only 6 out of 99 participants left the browser tab once in 16 trials, and no participant left the browser tab more than once. All six participants who left the browser tab once during the recognition task also did so at least once during the comparative judgment task. Therefore, we focused in our data checks on those participants who left the browser tab during the comparative judgment task. During the 140 trials of the comparative judgment task, 50.5% of participants never left the browser tab, 12% left the browser tab once, 24.2% left the browser tab between 2 and 5 times, 8.1% left the browser tab between 6 and 10 times, and 5.05% left the browser tab more than 10 times. The maximum number of times that a participant left the tab was 17. A single-trial analysis showed that all trials in which a participant left the browser tab resulted in correct comparative judgments. Therefore, we examined whether this had an effect on accuracy in the comparative judgment task. We found no correlation between the number of times a participant left the browser tab and the mean accuracy (*r* = 0.003, *p* = 0.97, BF_10_ = 0.21). We also found no conclusive evidence for an effect on accuracy in a comparison between participants who had left the browser tab at least once and participants who never did so (Welch test, *t*(96.8) = − 1.46, *p* = 0.14, BF_10_ = 0.55). Finally, we conducted the analyses (that we report in the subsequent “Results” section) after excluding those participants (*n* = 49 out of 99) who had left the browser tab at least once during the 140 trials of the comparative judgment task. Notably, we obtained largely the same results with this reduced dataset after following this strict exclusion criterion. Because exclusion of participants or individual trials did not affect our main conclusions, we included all participants and trials in the subsequent analyses. We nevertheless also report the results of the same analyses following the cautious strategy of excluding participants who left the browser (at the end of the “Results” section).

## Results

The abbreviations RR, RU+, RU−, UU refer to those trials in the comparative judgment task in which both city names were recognized (RR trials), in which only one city name was recognized (that was judged to have the higher criterion value: RU+; or not to have the higher criterion value: RU−), or in which both city names were unrecognized (UU trials).

### Recognition rate and applicability of the RH

On average, participants recognized *M* = 52.92% (*SD*= 10.6%) of the 15 cities used for the comparative judgment task. This resulted in a proportion of *M* = 51.14% cases (*SD* = 6.40%), where the RH was applicable (i.e., RU trials in which one of the two cities was recognized). Note that, recognition rate and applicability of the RH was identical between domains because they included the same pairs of city names.

### Comparative judgment task

Results of the comparative judgment task are shown in Table 2.^7^ The proportion of accurate judgments was higher in the population than in the distance domain, *t*(98) = 6.09, *p* < 0.01, *d* = 0.61, BF_10_ = 5.2 × 10^5^. Moreover, the mean recognition validity α was higher for judgments of city populations than of distances, *t*(98) = 21.80, *p* < 0.01, *d* = 2.19, BF_10_ = 1.1 × 10^36^; and the accordance rate to the RH was higher for judgments of city populations than of distances, *t*(98) = 9.85, *p* < 0.01, *d* = 0.99, BF_10_ = 2.45 × 10^13^. The knowledge validity β was slightly lower for judgments of populations than of distances, *t*(98) = − 2.40, *p* < 0.05, *d* = 0.24. Together, these results suggest that the judgment domains differed as expected with our manipulation. These findings are also largely in line with a previous study that used similar domains (Pohl 2006). To examine whether participants were able to adaptively adjust their reliance on the RH following these differences between domains, we next turn to the modeling results.

**Table 2:** Measures of the comparative judgment task as a function of domain (population vs. distance)

Measure	Ms		SDs	
	Population	Distance	Population	Distance
Proportion of accurate judgments	0.68	0.60	0.08	0.10
Choice of recognized object (RH accordance rate)	0.87	0.64	0.15	0.23
Recognition validity	0.77	0.45	0.08	. 09
Knowledge validity	0.62	0.68	0.18	0.16
RT_UU_	2421	3122	1645	3270
RT_RU+_	2222	3453	1940	6279
RT_RU−_	2770	3505	3425	6091
RT_RR_	2504	3410	2351	4434
Confidence in comparative judgment (UU)	25.26	24.75	18.09	18.62
Confidence in comparative judgment (RU−)	32.31	33.13	21.45	19.15
Confidence in comparative judgment (RU+)	53.26	43.61	18.58	20.84
Confidence in comparative judgment (RR)	55.16	54.92	17.40	19.95

*RH* recognition heuristic, *RU* trials in the inference task in which one of the two items was recognized, *RR* trials in which both items were recognized, *UU* trials in which both items were unrecognized, *RU*+ choice of recognized object, *RU*− choice of unrecognized object, *RH accordance rate* proportion of *RU* trials in which the recognized item is chosen, *RT* response time in ms

### Formal modeling: adaptive use of recognition

Table 3 shows the posterior means of the MPT *r*-model parameters and their standard deviations and correlations. Because the recognition validity is crucial for determining the usefulness of the RH in a given domain, we examined how participants adapted their reliance on recognition as a function of cue validity. In line with the environment-adaptivity hypothesis, we found a clear change in people’s use of the RH between domains: The RH was followed more likely in the population than the distance domain (model parameter *r*), Δ*r* = 0.57, 95% credibility interval: [0.44, 0.69], thereby following a corresponding change in the validity of recognition {Δ*a* = 0.32, [0.29, 0.34]}. Importantly, neither the validity of participants’ further knowledge {Δ*b* = − 0.03, [−0.07, 0.01]} nor the probability of making valid inferences on guessing trials (*UU* pairs) {Δ*g* = 0.02, [− 0.03, 0.07]} differed credibly between domains.

**Table 3:** Means, standard deviations, and correlations, of latent-trait model parameters (population-level posteriors)

Model parameter	Mean µ		Variability σ		Correlations ρ							
					*r*_POP_	*r*_DIST_	*a*_POP_	*a*_DIST_	*b*_POP_	*b*_DIST_	*g*_POP_	*g*_DIST_
*r*_POP_	0.79	[0.69, 0.87]	1.46	[1.17, 1.86]	1							
*r*_DIST_	0.22	[0.11, 0.33]	1.63	[1.30, 2.09]	[**0.15, 0.58**]	1						
*a*_POP_	0.77	[0.76, 0.79]	0.10	[0.01, 0.18]	[− 0.20, 0.74]	[− 0.29, 0.63]	1					
*a*_DIST_	0.45	[0.44, 0.47]	0.10	[0.02, 0.17]	[− 0.65, 0.26]	[− 0.48, 0.39]	[− 0.85, 0.26]	1				
*b*_POP_	0.64	[0.61, 0.67]	0.26	[0.18, 0.35]	[− **0.68**, − **0.17**]	[− 0.52, 0.07]	[− 0.66, 0.39]	[− 0.49, 0.54]	1			
*b*_DIST_	0.67	[0.64, 0.70]	0.31	[0.24, 0.38]	[− 0.26, 0.26]	[− 0.27, 0.26]	[− 0.37, 0.60]	[− 0.73, 0.14]	[− 0.32, 0.34]	1		
*g*_POP_	0.57	[0.53, 0.60]	0.31	[0.19, 0.43]	[− **0.76**, − **0.27**]	[− **0.63**, − **0.07**]	[− 0.74, 0.30]	[− 0.36, 0.66]	[**0.08, 0.73**]	[− 0.37, 0.31]	1	
*g*_DIST_	0.54	[0.51, 0.57]	0.19	[0.07, 0.30]	[− 0.15, 0.63]	[− 0.05, 0.67]	[− 0.44, 0.71]	[− 0.70, 0.38]	[− 0.57, 0.32]	[− 0.11, 0.72]	[− 0.77, 0.05]	1

Probability of reliance on recognition (model parameter *r*), validity of recognition (parameter *a*), and validity of knowledge (parameter *b*) as a function of task domain; *µ, σ*, and *ρ* refer to estimated population-level mean, standard deviation, and correlation, respectively, of the posterior model parameter estimates; 95% credibility intervals are in brackets; for the correlations, intervals that do not include zero are marked in boldface; the means of the latent-trait parameters are on the probability scale; standard deviations and correlations are on the probit scale

*POP* population domain, *DIST* distance domain

We also observed individual differences in all model parameters (none of the posterior intervals for the standard deviations in Table 3 includes zero), particularly in model parameter *r* (reliance on the recognition cue). However, does the variability in using the RH reflect individual differences in recognition validity, as the matching hypothesis suggests? An examination of the latent-trait correlations within domains provided little support for this possibility: Neither within the population domain {*ρ*_*r*POP*a*POP_ = 0.38 [− 0.20, 0.74]} nor in the distance domain {*ρ*_*r*DIST *a*DIST_ = − 0.06 [− 0.48, 0.39]}, did we find credible correlations among model parameters *r* and *a* (recognition validity). Interestingly, however, RH use was correlated across domains {*ρ*_*r*DIST*r*POP_ = 0.38 [0.15, 0.58]}, indicating some stability in people’s strategy preferences above and beyond the adaptive changes in RH use between environments.

**Fig. 2:**
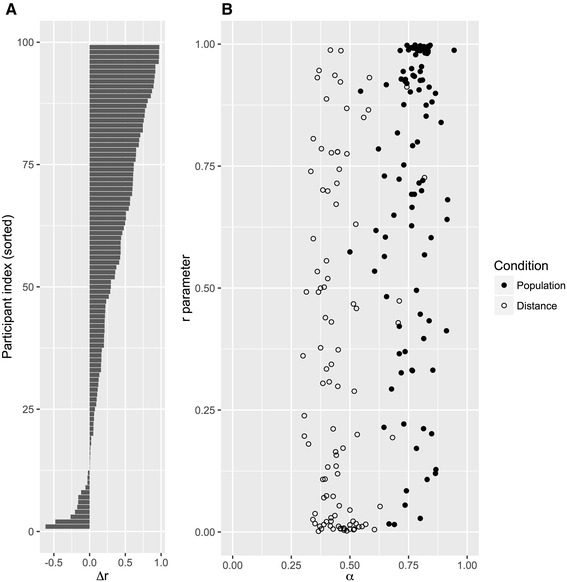
Adaptivity in recognition use at the individual level. **a** Change score of recognition use for each participant (difference in parameter *r* between domains, Δ*r* = *r*_POPULATION_ − *r*_DISTANCE_). A clear majority of participants uses the RH more likely when recognition validity is higher (in the population domain) but the magnitudes of this change are highly variable. **b** Model parameter *r* (reliance on recognition) plotted against the individual recognition validities (*α*) for the population and distance domains

In sum, a clear majority of individuals (*n* = 87 out of 99) adaptively changed their reliance on the RH, to varying degrees (see Fig. 2a). Notably, however, people did not necessarily follow their own recognition validities: their RH use and individual validities were uncorrelated (see Fig. 2b; Table 3 for correlations).

### Further measures of adaptivity: confidence reports

#### Metacognitive sensitivity

We next examined the role of confidence and its relation to adaptive strategy use. To this end, we first investigated potential differences in *metacognitive sensitivity* between the two task domains (population, distance) with a mixed logistic regression that models the relation of choice confidence and judgment accuracy. In this analysis, the slope parameter provides a measure of metacognitive sensitivity. The difference in slope between conditions as a function of confidence (i.e., the interaction term in the model), therefore, quantifies any potential differences in metacognitive sensitivity between domains. Notably, we found no significant interaction between confidence and domain, *χ*(1) = 0.16, *p* = 0.68, BF_10_ = 0.01, suggesting similar metacognitive sensitivity across domains. Based on this finding, we proceeded to compare the reported absolute confidence values between the two domains.

**Fig. 3:**
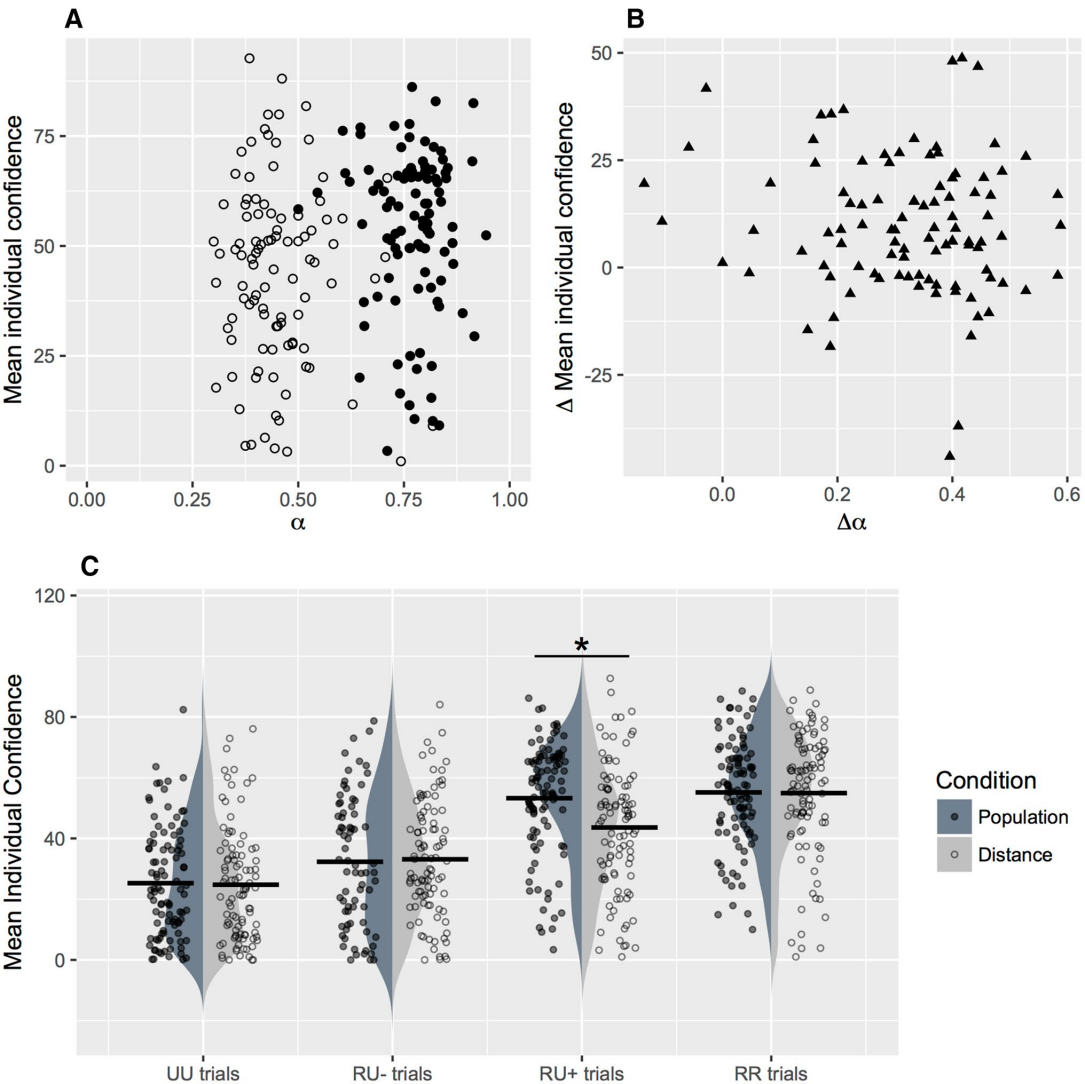
Confidence in the comparative judgments. **a** Mean individual confidence reports plotted against recognition validity α in each domain. **b** Difference in mean individual confidence between population and distance domains (for RU + trials, where the recognized city name is chosen) plotted against corresponding differences in *α*. **c** Distributions and group means (horizontal bars) of confidence reports as a function of trial type and domain (population, distance)

#### Individual confidence ratings

We compared individual confidence values across domains. To examine the implications of the matching hypothesis, we examined whether participants’ confidence tracked individual cue validities; that is, whether participants with higher α showed higher mean confidence on trials on which they followed the RH. To explore this possibility, we estimated the correlation between α and individual mean confidence on RU + trials (Fig. 3a). There was no correlation between α and confidence on RU + trials in the population domain, *r* = − 0.04, *t*(97) = − 0.44, *p* = 0.66, BF_10_ = 0.21, or in the distance domain, *r* = − 0.08, *t*(97) = − 0.83, *p* = 0.40, BF_10_ = 0.23. People may have systematic over- or under-confidence tendencies (e.g., Fischoff, 1982). Such tendencies, if present in our data, could introduce additional variance into confidence reports, thereby masking significant relations between α and mean confidence on RU + trials. To control for such tendencies, we also examined the correlation between intra-individual changes between domains in confidence and in corresponding *α* (Fig. 3b). Again, there was no evidence for a correlation between changes in α and in confidence between the two domains, *r* = − 0.12, *t*(97) = − 1.18, *p* = 0.24, BF_10_ = 0.12.

#### Mean confidence ratings

Finally, we examined mean confidence ratings across domains and trial types to evaluate the impact of task environment. Figure 3c shows confidence ratings for all trial types (RR, RU+, RU−, UU trials) as a function of domain (see also Table 3 for the means). We analysed these data in a 4 (trial type) × 2 (domain) ANOVA.^8^ First, there was a significant interaction between the trial types and domain on the confidence reports, *F*(3,216) = 8.521, *p* < 0.001, *η*^2^ = 0.106. That is, the effect of task domain on people’s confidence reports differed across the specific trial types in the comparative judgment task. Follow-up *t* tests revealed no differences in mean confidence on RR, UU, or RU − trial types between the population and the distance domains: all *t*s < 1.24, all BFs_10_ < 0.26. Notably, however, confidence ratings for RU + trials were significantly higher in the population than the distance domain (difference *M* = 7.68, *SD*  = 14.97), *t*(72) = 4.38, *p* < 0.001, *d* = 0.51, BF_10_ = 490. That is, confidence differences between domains emerged mainly on those judgment trials where the recognized object was chosen and the RH was applicable (RU + trials). Moreover, there was a significant main effect of trial type, *F*(3,216) = 140.2, *p* < 0.001, *η*^2^ = 0.661. The main effect of domain *F*(1,72) = 1.933, *p* = 0.169, *η*^2^ = 0.026 did not reach significance.

In sum, while participants did not show differences in metacognitive sensitivity between domains, we found differences in their (absolute) confidence ratings between domains, specifically on RU + trials. This suggests that recognition-validity differences between domains were available to metacognitive monitoring, even though differences in valid further knowledge and in criterion knowledge were negligible across these domains.

### Criterion knowledge

We used participants’ responses in the ranking task to estimate their criterion knowledge. For this purpose, we calculated the Spearman’s rank correlation for each domain between each participant’s ranking and the correct ranking of cities (for further discussion of the role of linear orders in inference, see, e.g., Schweickart & Brown, 2014, and Brown & Tan 2011). We then performed non-parametric tests on the individual rank-correlation values. We conducted two variants of this analysis. First, we restricted analysis to the cities recognized by each participant because it could be argued that criterion knowledge can only be meaningfully applied to cities that are recognized. However, Pohl and Hilbig (2012) suggested that unrecognized items might also be ordered. Therefore, we also repeated these analyses, including all city names. For recognized cities only, we found no differences in criterion knowledge between the population (*M* = 0.38, *SD* = 0.35) and the distance (*M* = 0.40, *SD*  =  0.41) domains (asymptotic one-sample permutation test: *T* = 19.82, *p* = 0.80; BF_10_ = 0.11). The similar levels of criterion knowledge in participants’ rankings suggest that the task difficulty was comparable across domains. However, including the unrecognized cities in the analysis revealed a significant difference in criterion knowledge between the population (*M* = 0.51, *SD*  =  0.17) and distance (*M* = 0.20, *SD*  = 0.31) domains (asymptotic one-sample permutation test: *T* = 33.4, *p* < 0.001, BF_10_ = 1.17 × 10^11^). These findings could suggest that for unrecognized city names (e.g., Fuyang), features such as their spelling or sound may nonetheless help people to make judgments of criterion values and that such features might be more helpful for judgments of the population than of the distance criterion.

### Supplemental analyses with exclusion of participants

We also repeated our analyses with a restricted sample of *N* = 50 participants who never left the browser tab during the experiment. This largely reproduced the results reported above: That is, there were again expected differences between the two domains in recognition validity *α, t*(49) = 16.28, *p* < 0.001, *d* = 2.30, BF_10_ = 2.09 × 10^18^, and in the reliance on recognition (*r* model parameter), *t*(49) = 5.70, *p* < 0.001, *d* = 0.81, BF_10_ = 2.3 × 10^5^.

Concerning confidence reports, the linear model again revealed no interaction effect, suggesting that metacognitive sensitivity did not differ between domains [*χ*^2^(1) = 2.03, *p* = 0.15, BF_10_ = 0.05]. Moreover, we found no correlations between α and mean confidence for either the population domain [*t*(48) = − 0.33, *p* = 0.74, *r* = − 0.05, BF_10_ = 0.37], the distance domain [*t*(48) = − 1.44, *p* = 0.16, *r* = − 0.20, BF_10_ = 0.51] or the change scores between the two [*t*(48) = − 0.24, *p* = 0.81, *r* = − 0.04, BF_10_ = 0.12]. The 4 (trial type) × 2 (domain) ANOVA on mean confidence ratings showed no significant interaction *F*(3,99) = 1.27, *p* = 0.29, *η*^*2*^ = 0.04. We nevertheless explored domain differences in follow-up *t* tests and again found that the confidence differences between domains on RU + trials remained [*t*(33) = 3.05, *p* = 0.004, *d* = 0.52, BF_10_ = 8.66] whereas there were again no significant confidence differences between domains on RR or RU − trial types (*t*s < 1.01; BFs_10_ < 0.29). Unlike the results with the full dataset, however, the confidence difference between domains in UU trials reached significance [*t*(33) = 2.13, *p* = 0.04, *d* = 0.37, BF_10_ = 1.36].

Finally, there were again no differences in criterion knowledge between domains (asymptotic one-sample permutation test *T* = 231, *p* = 0.10, BF_10_ = 0.16) when we included only the recognized cities. And, again, the same analysis including all cities indicated higher criterion knowledge for the population domain than the distance domain (*T* = 741, *p* < 0.001, BF_10_ = 8.67 × 10^5^). Taken together, the re-analyses with a restricted sample of participants largely confirmed the main conclusions reported for the whole sample.

## Discussion

In this study, participants made comparative judgments on a criterion dimension in two different task domains: In a population domain, where following recognition cues leads to relatively accurate judgments and in a distance domain, where this is unlikely. We observed that a clear majority of participants adaptively adjusted their reliance on recognition (albeit to highly varying degrees) and utilized recognition less frequently in a domain with lower recognition validity. We extended previous research by showing that this adaptive change occurs within individuals. This finding fills an important gap in the literature and could only be presumed so far on the basis of comparisons across group averages or across studies. Notably, one recent study found that individual differences in heuristic use of recognition are relatively stable across time, choice objects (different samples of city names), domains (success of celebrities vs. success of films), and presentation formats (verbal vs. pictorial), suggesting that people could have trait-like strategy preferences (Michalkiewicz & Erdfelder, 2016). The present findings are consistent with these reports, but highlight the complementary aspect of situation-specific, adaptive behavior. That is, we found that even under situations that differed substantially in recognition-cue validity, participants showed moderate stability in their reliance on recognition (as indicated by the positive correlation between the *r* parameters across the two different domains); importantly, above and beyond this stability, people showed flexible and adaptive behavior and substantially reduced their use of recognition in a situation where this was less useful.

It is an ongoing research issue to identify the processes leading to adaptive strategy selection. That is, why do participants utilize recognition more in contexts where it is more valid and how do they notice (i.e., on the basis of which information) when recognition cues are more valid? Based on previous theorizing, we reconsidered two possibilities in our within-subjects design: the matching hypothesis, which suggests that people are sensitive to their individual recognition validities; and the environment adaptivity hypothesis, which instead suggests that participants are only sensitive to more global differences between domains (e.g., Pachur et al. 2009; Pohl et al., 2017).

To test the *matching hypothesis*, we examined the covariation between individual RH use and recognition validities with a hierarchical multinomial model. The approach promised to sidestep possible biases involved in multi-step correlational analyses (Klauer 2010). We replicated previous findings and found no relationship between participants’ recognition use and the individual cue validities within the two domains (Pachur & Hertwig, 2006; Pohl 2006). Notably, and in extension of these findings, we also observed no relationship between individual changes in recognition use and corresponding cue validities across domains. Nonetheless, experience and frequent exposure to media could help people to develop intuitions about the usefulness of recognition. Hence, while direct access to the exact individual recognition validities appears unlikely, it is still possible that participants are somewhat sensitive to cue validities on a metacognitive level. Following this idea, we examined participants’ comparative judgments as well as their confidence in these judgments on each trial (reported on a visual analogue scale). The aim was to explore whether participants had some metacognitive intuition about their individual cue validities. We reasoned that if participants had metacognitive access to recognition validity, this would particularly influence their confidence on those trials where the recognized object was chosen. However, individual recognition validities were not associated with individual confidence ratings. Taken together, we found little evidence—both at the cognitive and metacognitive level—that participants’ judgments are guided by individual recognition-cue validities.

Participants showed adaptive and large changes in their reliance on recognition between the different task domains. Therefore, participants may have based their strategy use on more global factors, as the *environment adaptivity hypothesis* suggests. In this vein, we also examined changes in confidence between the domains. Confidence reports can be interpreted as a function of two separable factors: the strength of a monitored signal and a subjective criterion that determines the confidence level (Fleming & Lau, 2014). Hence, differences in confidence reports between domains could reflect differences in metacognitive sensitivity between domains (e.g., leading participants to inaccurately assign high confidence to erroneous judgments in one domain more frequently than in the other). Differences in confidence reports between domains could also reflect differences in the strength of the internal signal. The present findings suggest the latter, but provide no evidence for the former: We found no differences in metacognitive sensitivity between domains; differences in criterion knowledge or further knowledge were also subtle between domains. Nonetheless, we found clear differences in participants’ confidence ratings as function of domain as well as trial type.

Notably, the mean confidence ratings for judgments on RU + pairs and on *RR* pairs were similar in the population domain, but not in the distance domain. That is, when participants chose a recognized item in a domain with high recognition validity, even a lack of knowledge (only one name recognized) led to similarly high confidence as when both names were recognized. In other words, not recognizing a name contributed more to people’s confidence in the population than the distance domain. Moreover, in line with the notion that confidence is influenced by the most valid available cue (e.g., Gigerenzer et al. 1991), it is possible that participants’ confidence ratings on RU + trials were higher in the population than in the distance domain because the validity of recognition (*α* = 0.77) in the former was higher than the validity of further available knowledge (*β* = 0.68) in the latter. Overall, these findings are in line with the predictions of an extended environment adaptivity hypothesis and suggest that participants are also sensitive on a metacognitive level in which domain name recognition is more or less helpful.

## Limitations

Other perspectives and modeling approaches have been advocated to explain adaptive changes in judgment behavior. In network modeling, for instance, adaptivity has been conceptualized as a gradual change in the relative weight of various cues that are utilized for judgment (e.g., Glöckner et al. 2014) and not as a qualitative shift in strategy use (i.e., a shift in using the RH). Hence, the model parameter *r* in the present analyses could be alternatively interpreted as the degree of reliance on recognition, relative to other cues or information (see Heck & Erdfelder, 2017, for a discussion). Nonetheless, the present domain manipulations are informative from both modeling perspectives and provide a testbed for adaptive changes in judgment behavior. Moreover, the notion of qualitative strategy use (as assumed in the adaptive toolbox perspective) and of single-process mechanisms (e.g., in network modeling) is not necessarily mutually exclusive. For example, frugal lexicographic strategies have been fruitfully implemented as network models, too (Mata 2005). The different (modeling) perspectives on adaptivity discussed in the literature could mainly highlight different levels of analysis (Griffiths, Vul, & Sanborn, 2012).

Another potential limitation of the current study is that the data were collected online through the Mechanical Turk platform. In line with previous research that suggests compatibility between experimental online and laboratory studies (e.g., Crump et al. 2013), our results largely corroborated findings from a rich body of laboratory research on the RH (e.g., Hilbig et al. 2015; Marewski et al. 2010; Pohl 2006). However, some participants might have completed the current tasks in environments that were noisier than in typical laboratory settings. Approximately, 50% of the participants left the browser tab at least once during the task and might have searched for relevant information. This raises the issue of whether a city can still be considered as unrecognized if a participant read about it or whether a few participants have obtained some criterion knowledge during the experiment. Our data cannot fully exclude these possibilities, as we could not monitor or restrict what participants did outside the experimental task. However, we examined whether leaving the task had significant impact on accuracy, strategy use, or confidence ratings, and found little evidence for these possibilities. Moreover, we examined participants’ response times on individual trials, study duration times, and consistency between responses in different parts of the tasks, again providing little evidence that participants responded carelessly or that potential distractions had notable effects on our main conclusions.

Nonetheless, a potential avenue for future research could be a laboratory-based replication of the current findings.

## Conclusion

This study investigated within-person adaptivity in the frugal use of recognition for judgment. A clear majority of participants adaptively adjusted their strategy use between domains of different recognition cue validity. In line with previous studies that examined adaptivity between subjects, the use of recognition did not follow individual cue validities. Confidence reports suggested that participants assigned higher confidence to recognition in the domain with the higher recognition validity. Notably, this result could not be explained by differences between domains in people’s metacognitive sensitivity, in their criterion knowledge, or in their valid further knowledge, but instead suggests that people have good intuitions about global differences between judgment domains.

### Electronic supplementary material

Below is the link to the electronic supplementary material.

Supplementary material 1 (DOCX 273 KB)

## Figures and Tables

**Fig. 4:**
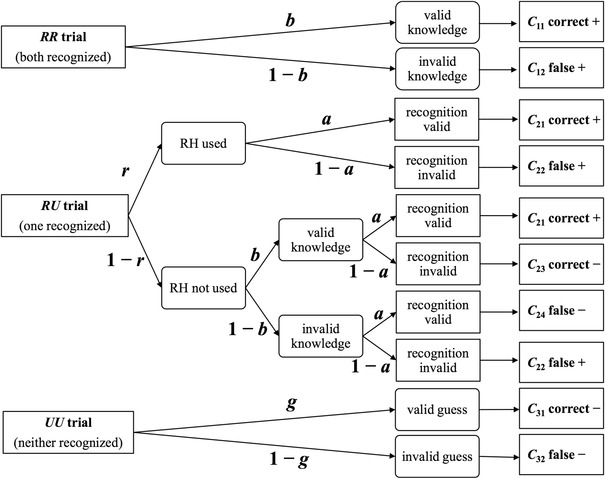
Illustration of the MPT r-model with parameters *b* (validity of further knowledge), *g* (probability of a correct guess), *r* (probability of reliance on recognition), and *a* (recognition validity)

**Fig. 5:**
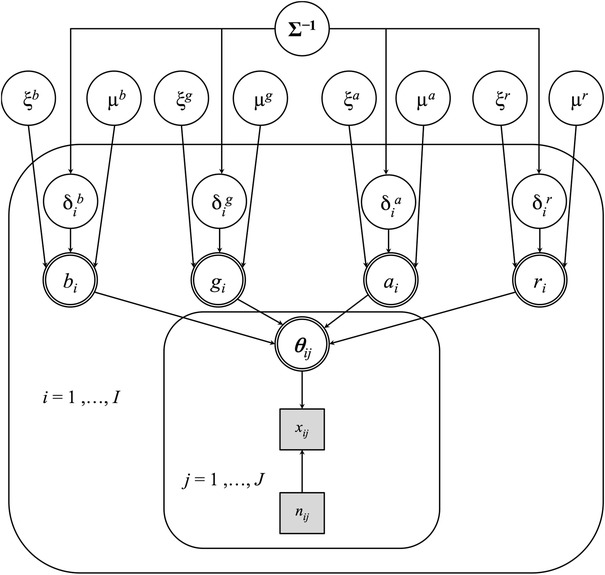
The graph structure illustrates a hierarchical latent-trait version (see Klauer 2010, and; Matzke et al. 2015, for further details) of the multinomial *r*-model, which is described in “Appendix A”. A model for one measurement domain is shown. Displayed are dependencies (i.e., probabilistic and deterministic relations) among latent model parameters and the data. Following conventional notation, observed variables are symbolized by shaded nodes, latent variables by unshaded nodes, continuous variables by circular nodes, and discrete variables by square nodes (Lee and Wagenmakers 2013). The plates indicate replications over the *J* = 3 different model trees (i.e., the three different trial types in the comparative judgment task: RR, RU, and UU cases) and over *I* individuals. For each individual *i*, the response data ***x***_*ij*_ (a vector with a participant’s category counts in a tree) follow a multinomial distribution with category probabilities $\varvec{\varTheta }$_*ij*_ and number of observations *n*_*ij*_, as defined in the *r*-model (see “Appendix A” for the tree structure)

## Appendix A

### Description of the multinomial processing tree model

Multinomial processing tree (MPT) models (see Batchelder & Riefer, 1999, and Erdfelder et al. 2009, for reviews) treat categorical response frequencies as probabilistic realizations of underlying cognitive states that are represented by model parameters. The MPT *r*-model (Hilbig, Erdfelder, & Pohl, 2010), used in the present analyses, can be illustrated in form of a tree diagram (Fig. 4).

The model accounts for three possible cases (trial types) in a comparative judgment task (i.e., RR, RU, and UU cases), represented by *J* = 3 separate trees. In each of the model trees, possible responses are assigned to one of the *K* mutually exclusive outcome categories *C*_*jk*_, distinguishing between inference accuracy (correct vs. false) and choice of recognized (+) versus unrecognized (−) items. In the context of the present analyses, the upper tree refers to the case where both city names are recognized (RR case), and therefore, further information (beyond mere name recognition) comes into play, leading to a correct inference with probability *b* and to an incorrect inference with complementary probability 1 − *b*. Parameter *b* thus indexes the validity of further information (beyond recognition) in conceptual equivalence to knowledge validity *β*. The second tree represents the case where only one of the two city names is recognized (*RU* case) and the RH can, therefore, be applied. With probability *r*, the decision-maker uses recognition as a cue and chooses the recognized name. This leads to a correct inference with probability *a* and to an incorrect inference with probability 1 − *a*. Parameter *a* measures the association between recognition and the criterion variable, in equivalence to the recognition validity α (Goldstein & Gigerenzer, 2002). With complementary probability 1 − *r*, the inference is based on further information beyond recognition (or any other strategy). This leads to a correct inference with probability *b*. The recognized object is then chosen with probability *a* and the unrecognized object is chosen with probability 1 − *a*. With probability 1 − *b*, the inference is incorrect. The unrecognized item is then chosen with probability *a* and the recognized item is chosen with probability 1 − *a*. In the bottom tree, neither city is recognized (UU case) and the decision-maker makes a correct inference with probability *g* (e.g., by making a guess). We employed a hierarchical Bayesian implementation of the r-model that accounts for individual variability and covariances among parameters. Further details are in “Appendix B”.

## Appendix B

### Graphical model of the hierarchical Bayesian implementation

The individual-level parameters **π**_*i*_ = (*b*_*i*_, *g*_*i*_, *a*_*i*_, *r*_*i*_) are modeled (in probit-transformed space) as π_i_ ← Φ (μ^π^ + ξ^π^·δ_*i*_^π^) and hence represent linear combinations of a group-level mean *μ*^π^ ~ *N*(0,1), a multiplicative scaling parameter, ξ^π^ ~ *U*(0,10), and an individual displacement parameter δ_*i*_^π^ that is drawn from a multivariate normal distribution with mean vector **0** and covariance matrix **Σ**^− **1**^ ~ Wishart (**I**, 5) prior distribution (Fig. 5). For the present purposes, we adapted the modeling code as described in Lee and Wagenmakers (2013) and in Matzke et al. (2015). In addition, we verified our results with the TreeBugs package for *R* by Heck, Arnold, and Arnold 2017.

## Appendix C

### Logistic mixed model analysis

We used mixed logistic regression models to quantify metacognitive sensitivity by comparing judgment accuracy for high-confidence trials vs. that for low-confidence trials. This method accounts for differences in confidence biases between participants (Rausch, Müller, & Zehetleitner, 2015; Wierzchoń, Asanowicz, Paulewicz, & Cleeremans, 2012). Logistic regression models describe the relationship between a binary outcome variable (in our case, response accuracy in the comparative judgment task) and several predictors (reported confidence). With mixed models, it is possible to directly analyze individual (and not the pooled) trials, while taking into account that multiple trials correspond to a single participant by assigning a different intercept and/or slope to each individual. Logistic regression models assume a linear relationship between the logarithm of the odds of the two possible outcomes and the modeled predictors (i.e., confidence) which is not always satisfied. For this reason, Rausch et al. recommended the use of other measures of metacognitive (“type 2”) sensitivity, such as meta-*d* (Maniscalco & Lau, 2012). These measures, however, rely on further assumptions of signal-detection theory (SDT). While there is ample evidence that well-controlled visual stimuli can be successfully modeled with SDT, this may not be warranted for comparative judgments, as in the present study. Therefore, we followed Siedlecka, Paulewicz, and Wierzchoń (2016) and operationalized metacognitive sensitivity as the slope of the mixed logistic regression model with judgment accuracy as the dependent variable and confidence as the predictor. We specified the model, including task domain (distance or population) and confidence, together with their interaction, as fixed effects. We also included individual city pairs (regardless of the presented order) and participant as random intercepts, and a random slope for the effect of confidence, thereby accounting for differences in metacognitive sensitivity between participants. We scaled and centered confidence as the only numerical predictor. We specified the models using the lme4 package for *R* (version 3.3.0) and report the *p* values from a likelihood-ratio test.

## Footnotes

1 The recognition validity α quantifies the predictive power of recognition and is conceptually similar to the ecological validity of a so-called “proximal cue” in Brunswik’s (1952) lens model. For a specific domain, it is defined as α = *C*_*RU*_ / (*C*_*RU*_ + *I*_*RU*_), where *C*_*RU*_ and *I*_*RU*_ are the correct and incorrect judgments, respectively, that would result from always choosing the recognized item in cases where *one* of the two items in a comparative two-alternative choice task is recognized (denoted as *RU* cases). Defined this way, α can range from zero to one; if α = 1, choosing the recognized item would always lead to a correct judgment; if α = 0.50, choosing the recognized object would lead to chance-level accuracy and recognition would be uncorrelated with the criterion dimension. The recognition validity is usually calculated for each individual and the averaged validities across individuals then serve as measure for the recognition validity in a specific task or domain (Goldstein and Gigerenzer 2002).

2 The reliance on recognition as a cue for inference in this section is reported as probability (i.e., ranging from 0 to 1), derived from model-based measures (Pohl et al. 2017).

3 Gigerenzer, Hoffrage, and Kleinbölting (1991) and Hoffrage (2011) extensively examined confidence reports in the context of cue-based decision making. These researchers found that when participants were not asked to evaluate a single judgment but were instead asked how many correct answers they gave (in a frequency format) people appeared well-calibrated and over-confidence effects disappeared, which speaks against the interpretation of overconfidence reflecting a general and stable “bias” in human judgment.

4 Scripts for the experiment are available in JavaScript under. https://zenodo.org/badge/latestdoi/99819531.

5 An analysis of task orders did not indicate significant sequence effects on using the recognition heuristic (further details are in the Supplemental Materials).

6 For MCMC sampling with JAGS (Plummer 2003), we ran three chains of 300,000 iterations each, discarded the first 200,000 iterations as burn-in, and used a thinning rate of 10. Chain convergence was satisfactory for all estimated model parameters (i.e., all $\widehat{R}$s < 1.0203; Lee and Wagenmakers 2013). For all model estimates, we report the medians of the MCMC samples. Further details about the modeling approach are in the Appendices and Supplemental Materials.

7 In addition to standard inferential indices and effect-size estimates, we report Bayes factors (*BF*_10_) to quantify the evidence for the alternative hypothesis relative to that for the null hypothesis. Several conventions exist to categorize the strength of evidence for a hypothesis. Following a classification by Jeffreys (1961), *BF*s_10_ between 1/3 and 3 indicate that the collected data are inconclusive (“anecdotal”); *BF*s_10_ < 1/3 indicate moderate-to-stronger evidence for the null hypothesis; *BF*s_10_ > 3 indicate moderate-to-stronger evidence for the alternative hypothesis.

8 Because some participants did not contribute any *RU* − trials (mainly in the population domain), this analysis excluded 26 participants with missing values in at least one cell. As an alternative analysis, in a 3 (trial type: *RU, UU, RR*) × 2 (domain) ANOVA, where trials were collapsed across *RU* + and *RU* − trials (and only one subjects’ data had to be discarded), we observed an interaction between trial type and domain, *F*(2,194) = 17.64, *p* < .001, η^2^ = 15), a main effect of trial type *F*(2,194) = 226.5, *p* < .001, η^2^ = 0.70, and a main effect of domain *F*(1,97) = 9.215, *p* = .003, η^2^ = 08.
