# Supplementary material for: Within-person adaptivity in frugal judgments from memory
Source: Psychol Res. 2017 Dec 22;83(3):613–30. doi: 10.1007/s00426-017-0962-7 (PMC6441105; doi:10.1007/s00426-017-0962-7)
Supplement: Supplementary file 1 — Supplementary material 1 (DOCX 273 KB) [file 426_2017_962_MOESM1_ESM.docx]

**Online Supplementary Information**

**Analysis of Sequence Effects**

We counterbalanced the order of the recognition and judgment tasks and the order of the domains and examined potential order effects on RH use (individual *r* parameters). In a 2 (domain order) × 2 (task order) × 2 (domain) mixed-model ANOVA, we found no effect of between-subjects factor domain order (population domain first vs. distance domain first), *F* < 1, *BF_10_* = 0.21, or between-subjects factor task order (recognition task first vs. comparative-judgment task first), *F*(1,95) = 1.10, *p* = .296, *BF_10_* = 0.36) but as expected, we found a significant effect of within-subjects factor domain (population vs. distance task; *F*(1,95) = 102.08, *p* < .01). We also found no significant interactions between domain and either task order, *F*(1,95) = 3.474, *p* = .06, *BF_10_* = .78, or domain order *F*(1,95) = 1.91, *p* = .17, *BF_10_* = .29.

**Congruency Index**

We computed a congruency index to estimate how consistently each participant responded across the comparative judgement and the ranking tasks, as both tasks require ordering the stimuli along the criterion dimension. To quantify response consistency across different tasks, we computed for each participant the proportion of trials in which the city chosen in the comparative judgement task had also been ranked higher in the ranking task, relative to the total number of trials in the comparative judgement task. In this way, a congruency index above 0.5 indicates an above-chance consistency between the two tasks for a given participant. None of the participants reached a congruency index of 1, but a substantial majority of participants displayed congruency indices above the value of 0.5 (that would be expected by chance). In both domains, the mean congruency index was significantly higher than 0.5. (Population: *M* = 0.8, *SD* = .09, *t(*98) = 30.57, *p* < .001, d = 3.07. Distance: *M* = 0.7, *SD* = .13, *t(*98) = 14.61, *p* < .001, *d* = 1.47).

**Data check: Easy “catch” trials**

We included two easy “catch” trials per domain, interleaved randomly within the 70 experimental trials. In the population domain, these trials compared the names of New York City vs. Detroit and Aspen vs. Miami. These trials were excluded from the main analyses reported above, and only analyzed separately as follows. We assumed that judgments for catch trials should be particularly easy and errors should mainly result from lapses of attention; indeed, 100% of participants provided an accurate judgment on both these trials. For the distance domain, the two additional easy pairs were Abu Dhabi vs. New York City and Bagdad vs. Detroit. All but 3 participants replied to these trials correctly (in all three wrong cases, New York City was chosen over Abu Dhabi as being closer to Dubai).

All in all, the data checks on focus on the task, congruency index analysis, and “easy” trials, together with the re-analyses (after removing potentially distracted participants) suggest that participants were carefully completing the tasks and provide support for the robustness of the conclusions.

**Fit of the Multinomial Processing Tree (MPT) Model**

The figure below provides a comparison of the hierarchical MPT model predictions and the observed data. Posterior predictive samples were generated from the latent-trait MPT model parameter estimates and the resulting distributions are visualized by boxplots. The observed mean response frequencies across participants in a category are marked by the blue circles. C1 to C16 refer to the categories as defined in the multinomial r-model that is described in Appendix A in the paper. Overall, the MPT model reproduced participants’ observed mean response frequencies across categories reasonably well. Graph created with the TreeBugs package for *R* by Heck, Arnold, and Arnold (2017).

**References Supplementary Information**

Bates, D., Maechler, M., Bolker, B., Walker, S. (2015). Fitting Linear Mixed-Effects Models Using lme4. Journal of Statistical Software, 67, 1-48. doi:10.18637/jss.v067.i01.

Heck, D. W., Arnold, N. R., & Arnold, D. (2017). TreeBUGS: An R package for hierarchical multinomial-processing-tree modeling. https://CRAN.R-project.org/package=TreeBUGS.
